# Polyelectrolyte-mediated increase of biofilm mass formation

**DOI:** 10.1186/s12866-015-0457-x

**Published:** 2015-06-06

**Authors:** Robert Bucki, Katarzyna Niemirowicz, Urszula Wnorowska, Marzena Wątek, Fitzroy J. Byfield, Katrina Cruz, Marta Wróblewska, Paul A. Janmey

**Affiliations:** Department of Microbiological and Nanobiomedical Engineering, Medical University of Bialystok, ul. Mickiewicza 2C, Bialystok, 15-222 Poland; Department of Physiology, Pathophysiology and Microbiology of Infections, The Faculty of Health Sciences of the Jan Kochanowski University in Kielce, Kielce, Poland; Department of Hematology, Holy Cross Oncology Center of Kielce, Kielce, Poland; Department of Physiology and the Institute for Medicine and Engineering, University of Pennsylvania, Philadelphia, PA USA; Department of Dental Microbiology, Medical University of Warsaw, Warsaw, Poland

**Keywords:** Bacteria, *C. albicans*, Filamentous bacteriophages, DNA, F-actin, Biofilm mass

## Abstract

**Background:**

Biofilm formation is associated with various aspects of bacterial and fungal infection. This study was designed to assess the impact of diverse natural polyelectrolytes, such as DNA, F-actin, neurofilaments (NFs), vimentin and purified Pf1 bacteriophage on biofilm formation and swarming motility of select pathogens including *Pseudomonas aeruginosa* associated with lung infections in CF patients.

**Results:**

The bacteriophage Pf1 (1 mg/ml) significantly increased biofilm mass produced by *Pseudomonas aeruginosa* P14, *Escherichia coli* RS218 and *Bacillus subtilis* ATCC6051. DNA, F-actin, NFs and Pf1 also increased biofilm mass of the fungal *C. albicans* 1409 strain. Addition of F-actin, DNA or Pf1 bacteriophage to 0.5% agar plates increased swarming motility of *Pseudomonas aeruginosa* Xen5.

**Conclusions:**

The presence of polyelectrolytes at infection sites is likely to promote biofilm growth and bacterial swarming.

## Background

Bacterial biofilm, a complex community of surface-associated cells surrounded by a self-produced polymer matrix consisting mostly of polysaccharides and DNA can form at infection sites and promote chronic infection [1–3]. Biofilm increases bacterial resistance to the host immune system, provides a place for development of resistant microorganisms and is associated with increased resistance to exogenous antibiotic treatment [4–6]. Therefore, once established, biofilm infections are nearly impossible to eradicate [7]. Biofilm formation involves the production of an extracellular matrix, but the composition of this matrix is not well defined. In *Pseudomonas aeruginosa* PA14, decreased production of extracellular polymers reduces static biofilm development [8]. An increasing number of studies support the hypothesis that DNA, F-actin, and possibly other filamentous polymers released from host inflammatory cells, as well as products of bacterial autolysis, trigger genetic changes in planktonic bacteria that initiate the formation of biofilm [9, 10]. Extracellular DNA and F-actin have been shown to function as a structural support for bacterial biofilm architecture [4, 11, 12] and have been proposed as therapeutic targets to prevent biofilm growth [5, 13]. Culturing *Pseudomonas aeruginosa* (PA) in settings that include neutrophils enhances formation of biofilms by stimulating the expression of alginate [5]. Recently, extracellular DNA was found to smooth the progress of efficient traffic flow of PA cells throughout the complex matrix of biofilm by maintaining coherent cell alignment, thereby avoiding traffic jams and ensuring an efficient supply of cells to the migrating front [14]. However, biofilm formation and swarming by clinical isolates of PA were found to be negatively associated [15]. Another consequence of negatively-charged polymers such as DNA and F-actin present at infection sites results from their ability to bind and inactivate host-derived cationic antibacterial molecules [16, 17]. Therefore, poly-anionic filamentous viruses produced by *Pseudomonas*, which are important for the virulence of these pathogens in humans, might inactivate host antibacterial molecules by similar mechanisms. The PA strain PAO1 strongly up-regulates genes encoding Pf1-like phages at the start of biofilm formation, and Pf1-like virions are present at 100 to 1000 fold higher levels in biofilms when compared with planktonic PA cultures [18]. The Pf1 phage exists largely as bundles of long filamentous phages extending from the bacterial surface [19]. Bundle formation was previously found to be mediated by polyvalent counterions, including cationic host defense molecules [20]. A repeatable pattern of cell death and lysis that occurs in biofilms during the normal course of development is also phage-dependent, and phage-mediated cell death is an important mechanism of differentiation inside microcolonies that facilitates dispersal of a subpopulation of surviving cells [21]. This report indicates that addition of diverse natural polyelectrolytes, including purified Pf1 bacteriophage, to different bacteria or *Candida albicans* cultures results in increased biofilm formation. The effects of Pf1 on biofilm formation by bacterial strains that it cannot infect suggests that the physicochemical properties of Pf1 and similar polyelectrolytes, rather than any specific biological effect of the bacteriophage on the bacteria is required for it to support biofilm formation by different microorganisms.

## Results and discussion

### Polyelectrolyte-induced formation of microorganisms’ biofilm

**Fig. 1:**
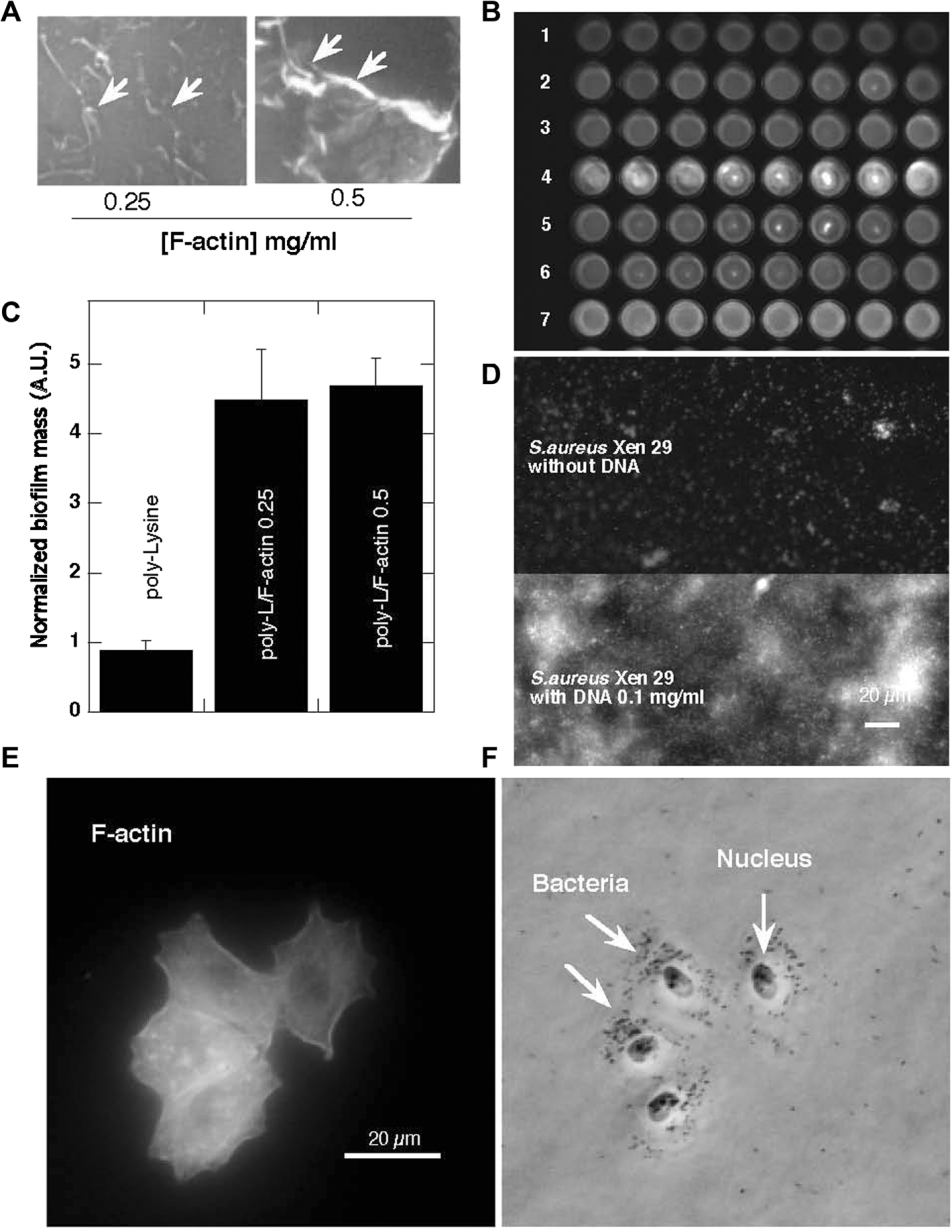
Bacterial biofilm formation in the presence of F-actin and DNA. **a** Arrows indicate F-actin filaments stained with rhodamine-phalloidin and attached to a glass slide coated with poly-Lysine (0.1% water solution). **b** Biofilm mass of *Pseudomonas aeruginosa* PAO1 formed in the presence of F-actin. **c** Chemiluminescence of *Pseudomonas aeruginosa* Xen5 48 h after bacteria addition to LB broth (row 1) containing F-actin (rows 2, 3, 4) or DNA (rows 5, 6, 7) at concentrations of 0.01, 0.05 and 0.1 mg ml ^−1^ respectively. **d** DNA staining (YOYO-1) of *Staphylococcus aureus* Xen29 biofilm after 48 h growth with and without the presence of DNA. **e** F-actin filaments of lung epithelial A549 cytoskeleton stained with rhodamine-phalloidin (cells were treated with 0.01% Triton X-100 for 10 min and washed 3x with PBS before staining). **f** *Pseudomonas aeruginosa* PAO1 cells preferentially attached to the cytoskeleton of A549 cell remnants

**Fig. 2:**
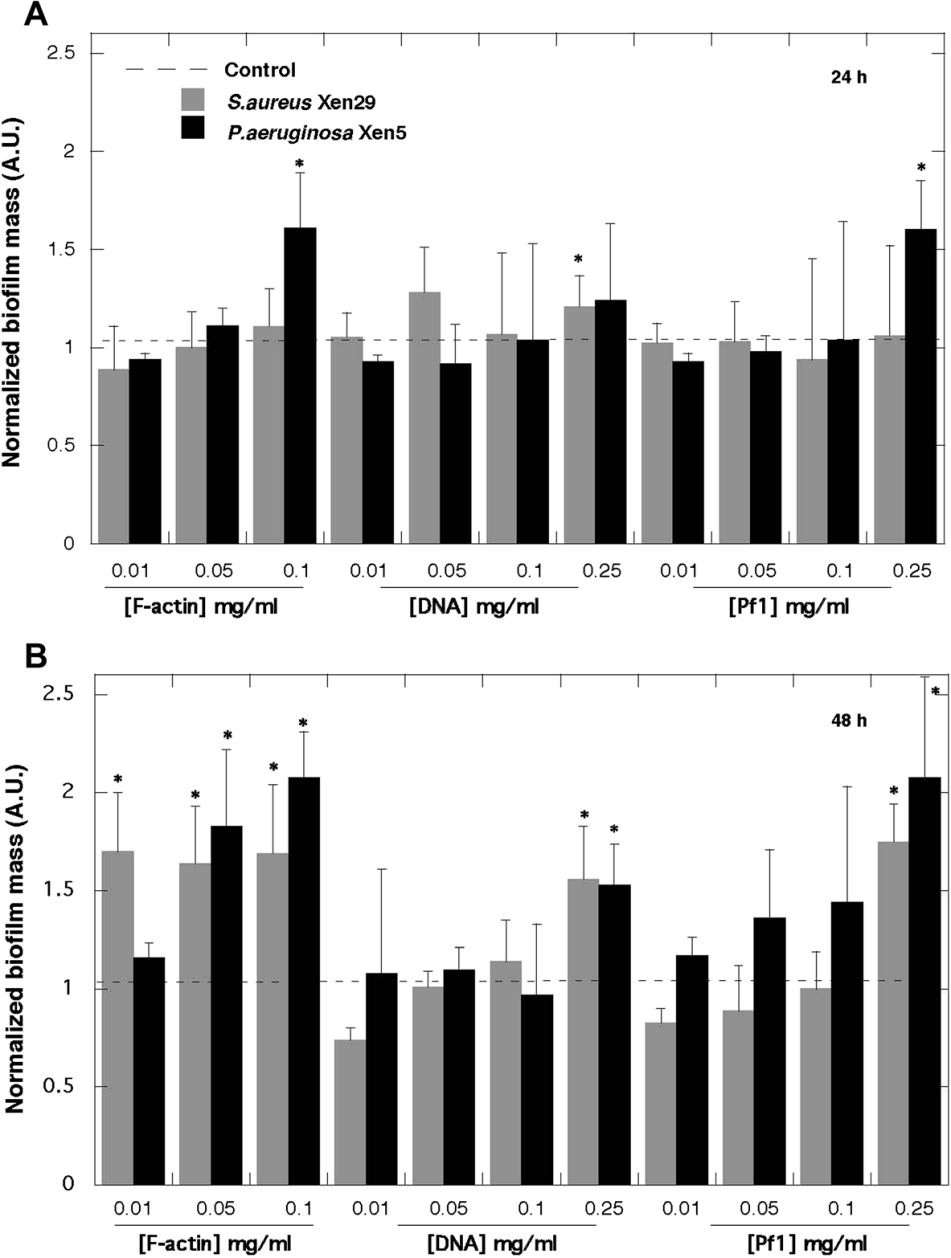
F-actin, DNA and Pf1 bacteriophages increase *Staphylococcus aureus* Xen29 and *Pseudomonas aeruginosa* Xen5 biofilm formation. Panels **a** and **b** indicate biofilm mass assessed with crystal violet staining at 24 and 48 h time points. *Significantly increased compared to control

**Fig. 3:**
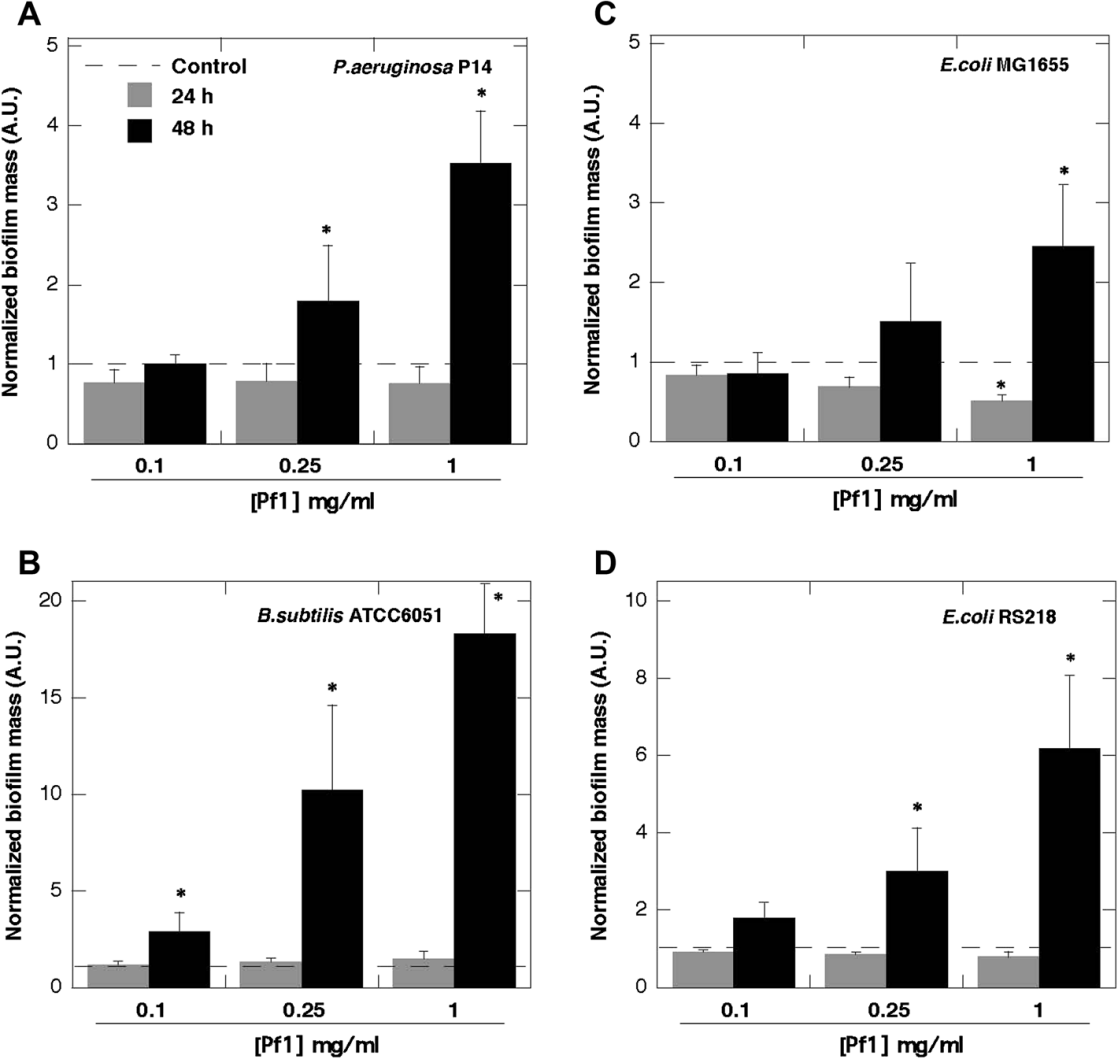
Pf1-mediated increase of biofilm formation. Biofilm formation of *Pseudomonas aeruginosa* P14 (panel **a**), *Bacillus subtilis* ATCC 6051 (panel **b**), *Escherichia coli* MG1655 (panel **c**) and *Escherichia coli* RS218 (panel **d**) in the presence of Pf1. Biofilm mass was assessed using crystal violet staining at 24 and 48 h time points. *Significantly increased compared to control

In agreement with previous reports [5, 13] F-actin increased biofilm formation by PA PAO1 on glass (Fig. 1a and b). Increased chemiluminescence of PA Xen5 bacteria culture after 48 h growth in the presence of F-actin or DNA was observed (Fig. 1b: rows 1-control, 2,3,4- increasing concentration of F-actin; 5,6,7—increasing concentration of DNA). DNA staining with YOYO-1 revealed an increased concentration of DNA in *S. aureus* Xen29 biofilm formed in the presence of exogenous DNA (Fig. 1d). This observation suggests that bacteria might use their own and likely other species’ extracellular DNA to form a biofilm matrix. Increased attachment of PA PAO1 to the cytoskeleton fraction of A549 cells attached to the coverglass surface after Triton x-100 treatment indicates that bacteria are able to bind the F-actin network (Fig. 1e and f). Compared to DNA and Pf1, F-actin has a stronger stimulatory effect on biofilm mass of PA Xen5 and *S. aureus* Xen29 (Fig. 2). Among all tested bacteria strains, the largest increase in biofilm production was observed after 48 h for *B. subtilis* growth in the presence of 0.25 and 1 mg/ml Pf1 (p < 0.001) (Fig. 3b). A statistically significant increase in biofilm formation was also observed at 48 h in PA P14 culture after addition of 0.25 and 1 mg/ml Pf1 (p < 0.05 and p < 0.001 respectively), in *E coli* MG1655 culture with addition of 1 mg/ml Pf1 (p < 0.001) and *E.coli* RS218 culture with addition of 0.25 and 1 mg/ml Pf1 (p < 0.05 and p < 0.001 respectively) (Fig. 3). The observed differences between the amounts of biofilm formed in the presence of Pf1 bacteriophage for different bacterial strains might be a result of growth rate differences.

**Fig. 4:**
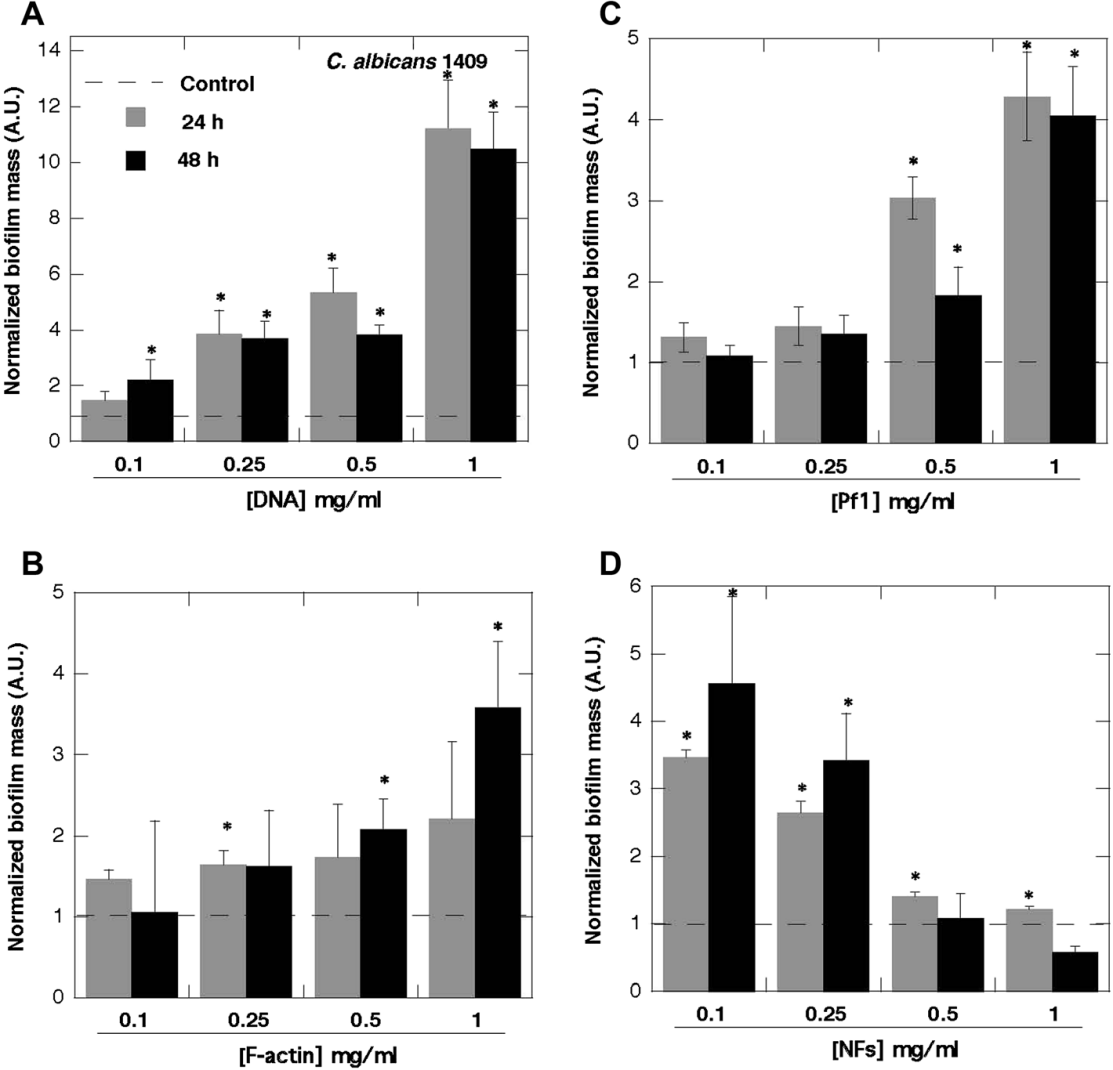
Biofilm formation of *C. albicans*. Increase of *C. albicans* 1409 biofilm formation in the presence of DNA (panel **a**), F-actin (panel **b**), Pf1 (panel **c**) and NFs (panel **d**) (*n* = 6). *Significantly different compared to control

DNA, F-actin, neurofilaments and vimentin are all anionic polyelectrolyte filaments present within the cellular and extracellular space, that have large net negative charge densities distributed over their surfaces and all promote biofilm formation (Figs. 1, 2 and 3 and data not shown). However, there are some differences between their activity that may result from filament properties or the growth of different bacteria strains. Neurofilaments have little effect on PA Xen5 biofilm formation compared to their effect on *S. aureus* Xen29, which is more than doubled in the presence of neurofilaments at a concentration of 0.1 mg/ml. Vimentin slightly increases biofilm formation of both PA Xen5 and *S. aureus* Xen29 (data not shown). Polyelectrolyte-induced biofilm formation was also observed for the fungus *C. albicans* 1409, which is a frequent cause of fungal infection in humans. The fungal biofilm matrix is mostly produced by the fungal cells themselves and is composed of different types of biopolymers [22]. As shown in Fig. 4, DNA, Pf1, F-actin and NFs all increase the formation of *C. albicans* biofilm. Among these polyelectrolytes, the strongest activity was observed for DNA, Pf1, F-actin, and NFs (Fig. 4). At the tested concentration range (0.1-1 mg/ml) NFs reveal the potential to promote *C. albicans* biofilm formation only at low concentrations. At a concentration greater than 0.5 mg/ml, NFs decrease the amount of biofilm formed, an effect that might be related to their high negative charge density. At a high concentration NFs might function as neutrophil extracellular traps (NETs).

### Increase of *P. aeruginosa* swarming activity in the presence of polyelectrolytes

**Fig. 5:**
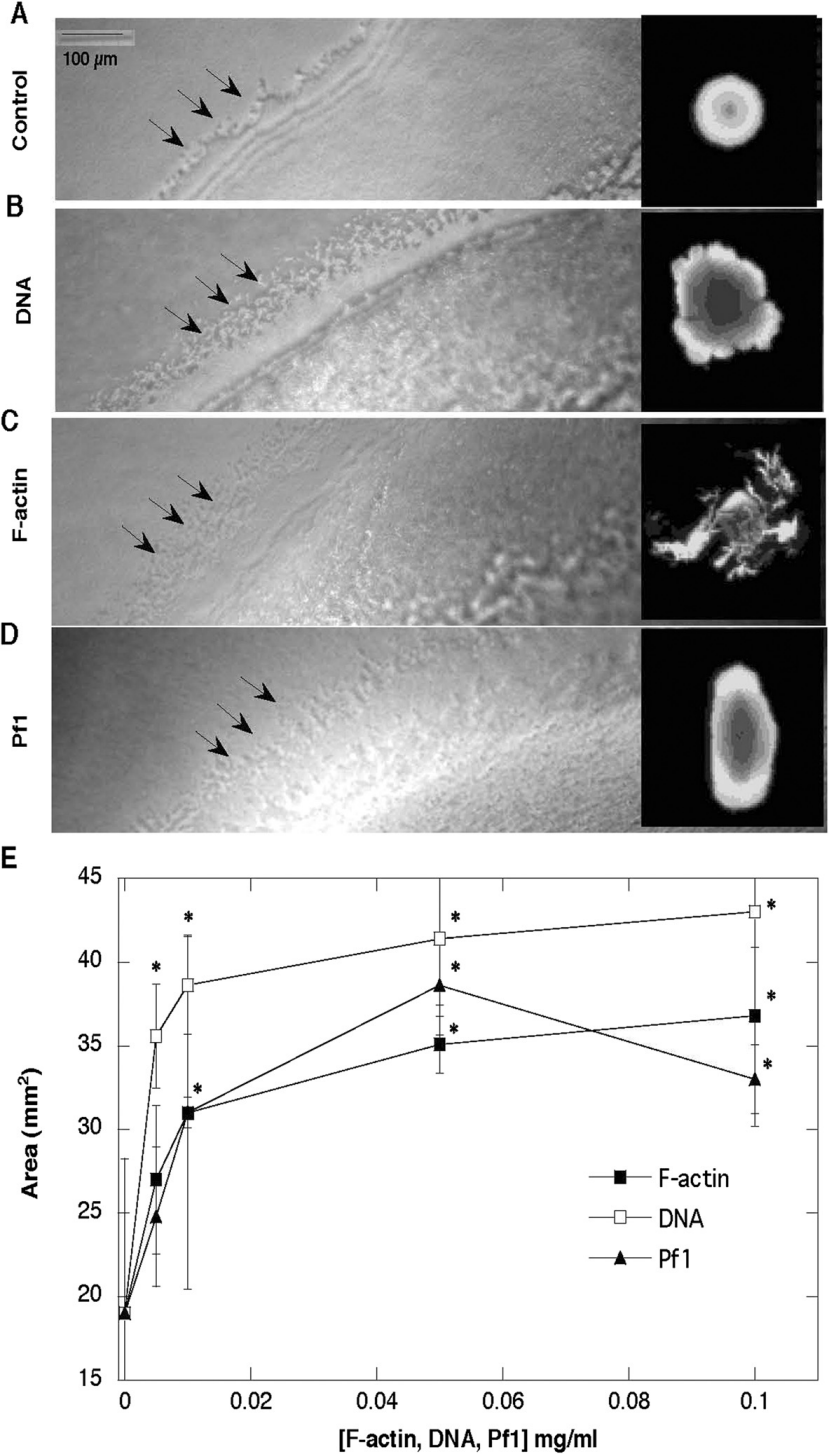
Morphology of *Pseudomonas aeruginosa* Xen5 biofilm growth on agar with addition of DNA, F-actin and Pf1 bacteriophage. In the presence of different biopolymers (0.1 mg/ml), after 16 h of bacterial growth, the swarming edges show some similar features (they are more diffuse and less defined; panel **a**-**d**). Developing area of spreading PA biofilm due to swarming activity was assessed using Image Gauge software (panel **e**). *Significantly different compared to control

Bacterial swarming motility and production of exopolysaccharides both contribute to biofilm formation, yet it is unclear how bacteria coordinate these two processes [23]. For some bacteria, the ability to swarm positively correlates with production of exopolysaccharides [24]. In contrast, alginate oligomers (OligoG) were found to bind to the bacterial surface, modulate surface charge, induce microbial aggregation, and inhibit swarming motility [25]. Figure 5 shows the morphology of a *P. aeruginosa* Xen5 biofilm that develops in the presence of different biopolymers. At the macroscopic level, a more fragmented pattern of growth was observed in the presence of F-actin, compared to uniform growth in DNA- or Pf1-containing samples. As indicated by the arrow in Fig. 5, on the edges of the biofilm where the swarming assures spreading of bacteria, more rounded structures less than 10 μm in size are observed in substrates containing biopolymers compared to control substrates. Quantitative analysis of biofilm indicates that all tested biopolymers increase swarming activity of PA Xen5. Among these biopolymers, there is a higher average increase of swarming area for substrates containing DNA than F-actin or Pf1 at the higher concentrations (0.1 mg/ml). In agreement with our study, recent results indicate that among the tested swarming mutants, a large range of biofilm formation levels was observed [26].

### Lower mass of biofilm formed in the presence of DNase I

**Fig. 6:**
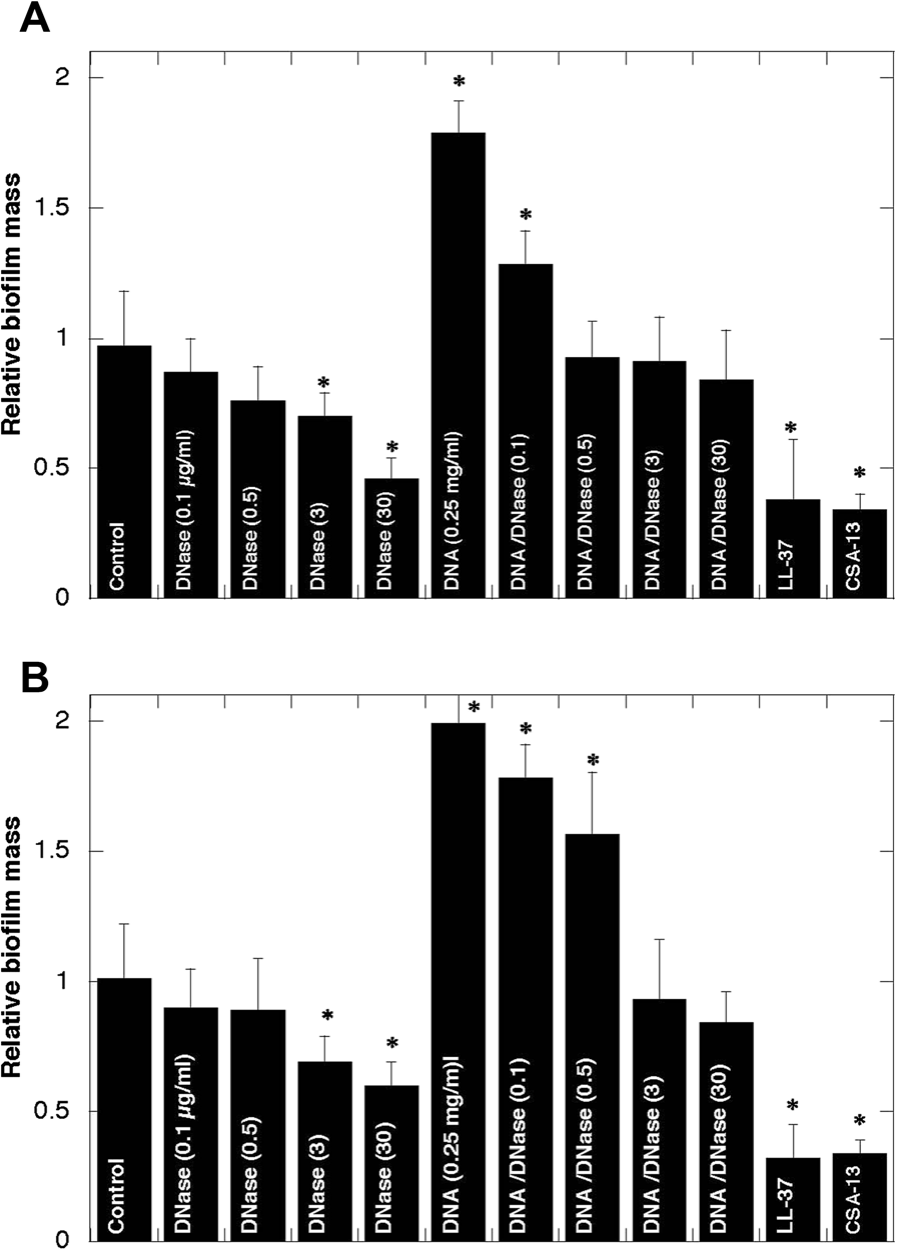
Biofilm formation in the presence of DNA and DNase I*. Pseudomonas aeruginosa* Xen 5 (panel **a**) and *Staphylococcus aureus* Xen 29 (panel **b**) biofilm at 48 h in 50% LB with DNA 0.25 mg/ml in the absence/presence of DNase I (0.1–30 μg/ml) and in the presence of LL-37 or CSA-13 (20 μM each). Inhibitory effect of DNase was compared to the effect of CSA-13 and LL-37 (*n* = 4). *Significantly different compared to control

Two clinically important biofilm-forming bacterial pathogens, PA and *S. aureus* were used to assess the effects of DNAse I and cationic antibacterial agents on biofilm mass. Addition of Pulmozyme (DNAse I) at 0.5 μg/ml significantly decreases the biofilm mass of both pathogens grown for 48 h with and without DNA (p < 0.01). In an earlier study, the addition of poly(aspartic acid) was found to reduce adhered microflora and decrease dental plaque formation [27]. The combination of DNase I and poly(aspartic acid) decreased PA biofilm formed in the presence of activated neutrophils by 79.2% on hydrogel contact lenses [13], and disrupted mature PA biofilm [4]. In our experimental settings, poly(aspartic acid) decreased biofilm formation but the effect did not reach a statistically significant level (data not shown). This difference might potentially be associated with the length of polymer used. On the other hand, in accordance with previous observations that the polycationic antimicrobial peptide LL-37 blocks biofilm formation at concentrations below its MIC, [28] decreased biofilm mass of both pathogens in the presence of LL-37 peptide (p < 0.001) was observed. A similar decrease was found in the presence of the cationic antibacterial steroid ceragenin CSA-13 (Fig. 6).

## Discussion

Accumulation and death of neutrophils recruited in response to pathogen invasion and tissue destruction release various negatively-charged biopolymers including DNA, vimentin, and F-actin. Destruction of neuronal cells can be expected to release NFs in addition to F-actin and DNA, whereas damaged muscle cells might predominantly release actin. Bacteria also support the accumulation of extracellular anionic biopolymers since they can release exopolysaccharides, bacterial DNA, as well as different filamentous phage. A growing body of evidence indicates that these biopolymers can interfere with host immunoresponse to infections. Extracellular DNA (eDNA) is a major structural component in many biofilms of pathogenic bacteria [1, 29]. Additionally, the functional DNA binding-uptake system that can be inhibited by the presence of DNase l [9], DNA-induced changes in gene expression [30], and sphingomyelinase-mediated cross-linking of eDNA in the biofilm skeletal framework [31] are all involved in biofilm formation by different bacterial pathogens. Results of our study indicate that the ability to stimulate biofilm formation might be a common feature of negatively charged biopolymers, including Pf1-like phages expressed by PA strains preferentially infecting CF patients. However, there are conflicting interpretations as to whether production of the filamentous phage virions encoded within *P. aeruginosa* as they colonize the airway increase the virulence of *P. aeruginosa* strains. The increased production of biofilm in the presence of Pf1 suggests that phage expression should be considered as a factor that increases the virulence of *Pseudomonas*.

Determining the relevant concentration range of phage and other polyelectrolytes is guided by several considerations. Infected, purulent body fluids such as cystic fibrosis sputum can contain as much as 50 mg ml^−1^ DNA and 10 mg ml^−1^ F-actin. Since Pf1 is not lytic and diffuses very slowly from its site of production, the relevant virus concentration is that adjacent to the bacteria, as seen in [19]. Quantitative studies of the rate or extent of Pf1, Pf4, or Pf5 production by *Pseudomonas* strains have not yet been reported, but *E. coli* can produce 830 copies of the structurally similar 800 nm long filamentous phage M13 per hour [32]. Making the conservative estimate that a 0.5 μm x 2 μm cylindrical *Pseudomonas* produces 800 viruses extending from the bacterial surface, and using the fact that a polymer of length 2 μm and persistence length 2 μm has an end-to-end distance of 1.7 μm [33], the local concentration of Pf1 within the zone 1.7 μm away from the bacterial surface is approximately 1 mg ml^−1^. This concentration is within the range at which Pf1 promotes biofilm growth. Therefore [Pf1] between 0.1 and 5 mg ml^−1^ is likely to be relevant to a purulent setting. These findings also indicate that the physicochemical properties of filamentous bacteriophage, and its concentration, rather than the ability of Pf1 to infect and propagate in bacteria, are sufficient to promote biofilm growth. Pf1 has a very similar size, shape, and charge density as F-actin and aggregates similarly with multivalent cations. These features might explain the strong ability of both polymers to induce biofilm formation [5, 34], as well as that of neurofilaments and vimentin [35]. In agreement with recent studies showing an increase of *C. albicans* biofilm formation induced by its own DNA as well as DNA released from bacteria [12, 36], we observed that DNA and other natural polyelectrolytes such as F-actin, Pf1 and NFs can all stimulate this process. The dose-dependent effect recorded for NFs (concentrations above 0.5 mg/ml resulted in decreased biofilm formation) was previously reported for DNA [36].

The effect of anionic biopolymers to promote biofilm formation may depend on their effect on swarming mobility. Many bacteria move in groups, in a mode described as swarming to colonize surfaces and form a biofilm to survive external stresses, including exposure to antibiotics [37]. Considering the possibility of charge-driven interactions, antibiotic resistance might result from the ability to sequester positively-charged antibiotics by negatively-charged polymeric components [17, 38] of the biofilm network before the antibiotics reach and insert into bacterial cells. This mode of bactericidal agent inactivation affects natural host defense cationic peptides such as cathelicidin LL-37.

## Conclusion

The presence of polyelectrolytes at infection sites such as DNA, F-actin, neurofilaments and Pf1 bacteriophage is likely to promote biofilm formation. However, the mass of developing biofilm in the presence of the same kind of polyelectrolytes varies among different microorganism strains.

## Methods

### Materials

Cetrimide Agar (Pseudomonas selective agar base) and deoxyribonucleic acid from herring sperm were from Sigma. Luria-Bertani broth (LB) and tryptic soy broth (TSB) were from DIFCO (Sparks, MD). Dulbecco’s Modified Eagle media (DMEM) was from GIBCO, (Grand Island, NY). Fetal bovine serum was from Hyclone, (Logan, UT). PA Xen 5 and *S. aureus* Xen29 strains engineered through conjugation and transposition of a plasmid carrying transposon Tn*5 luxCDABE* were purchased from Caliper Life Science Inc. (CA, USA). *B. subtilis* ATCC6051, *E. coli* MG1655, *E. coli* RS218 and *C. albicans* 1409 was purchased from ATTC collection and Polish Collection of Microorganism (PCM). High affinity nucleic acid stain YOYO®-1 was from Life Technologies (Grand Island, NY). Monomeric G-actin was prepared from acetone powder of rabbit skeletal muscle [39] and kept in non-polymerizing solution contained 2 mM TRIS, 0.2 mM CaCl_2_, 0.5 mM ATP, 0.2 mM DTT pH 7.4. Actin polymerization was induced by adding 150 mM KCl and 2 mM MgCl_2._ Neurofilaments (NFs) were isolated from bovine spinal cord according to procedures described previously [40]. Protease free, Pf1 bacteriophage strain LP11-92 that was propagated in *P. aeruginosa* LA23-99 was from ASLA Biotech Ltd. (Riga, Latvia).

### Cell Culture

Human lung epithelial carcinoma cells, line A549, were cultured in DMEM supplemented with 10% fetal bovine serum at 37 °C with 5% CO_2_. Cells were seeded and allowed to spread for 24–72 h before experiments were performed. A549 cells were fixed with 4% paraformaldehyde (Sigma-Aldrich) for 10 min at room temperature and subjected to 0.1% solution of TRITON X-100 treatment to expose the cytoskeleton F-actin network. When required, staining with 1:100 rhodamine-labeled phalloidin (Invitrogen) in PBS for 30 min was performed. To assess bacterial ability to interact with the cytoskeleton, a suspension of PA PAO1 (10^5^ CFU/ml) was added to A549 cell remnants for five minutes. Fluorescence and bacterial attachment were evaluated after 3 washes with PBS, using a LEICA DM IRBE. Images were captured with a Cool SNAP (HQ) camera (Trenton, NJ, USA).

### Biofilm formation

The mass of biofilms was assessed using Crystal Violet (CV) staining (0.1%) [41] and chemiluminescence intensity measurements as an additional determinant of biofilm viability [42]. In each experiment an overnight culture of *P. aeruginosa* Xen 5, *P. aeruginosa* PAO1, *P. aeruginosa* P14 in TSB or *S. aureus* Xen 29, *B. subtilis* ATCC6051, *E. coli* MG1655, *E. coli* RS218 and *C. albicans* 1409 in LB was diluted to ~ 10^5^ CFU/ml. When required, bacterial suspensions were placed on glass slides coated with poly-Lysine/F-actin or in flat bottom PVC microplates (MP Biomedicals; Solon, OH) sealed with a gas permeable membrane (USA Scientific; Ocala, FL). Bacteria or *C. albicans* were grown in 50% LB broth with or without F-actin, DNA, NFs, vimentin or Pf1 bacteriophage. A stationary biofilm was allowed to form for 24 or 48 h. PA Xen5 and *S. aureus* Xen29 chemiluminescence signals were evaluated using a Fuji Film LAS-300 system. Densitometry analysis was performed using Image Gauge (version 4.22) software.

### *Pseudomonas aeruginosa* Xen5 swarming motility

A swarming motility assay was assessed as described previously [43]. Briefly, 3 μl of bacterial inoculum containing ~10^8^ CFU/ml were placed in the center of 0.5% agar containing modified M9 medium with different concentrations of F-actin, DNA or Pf1 bacteriophage (0.005–0.1 mg/ml) in 6 well plates. Swarming area was evaluated from images captured with a Fuji Film LAS-300 system analyzed with Image Gauge (version 4.22) software.

### Statistical analysis

Data are reported as mean ± SD from 3–8 repeats. Differences between means were evaluated using the unpaired Student’s *t*-test and differences p < 0.05 were considered statistically significant.
